# Kite Aerial Photography for Low-Cost, Ultra-high Spatial Resolution Multi-Spectral Mapping of Intertidal Landscapes

**DOI:** 10.1371/journal.pone.0073550

**Published:** 2013-09-19

**Authors:** Mitch Bryson, Matthew Johnson-Roberson, Richard J. Murphy, Daniel Bongiorno

**Affiliations:** 1 Australian Centre for Field Robotics, The University of Sydney, Sydney, NSW, Australia; 2 The Department of Naval Architecture and Marine Engineering, University of Michigan, Ann Arbor, Michigan, United States of America; National Institute of Water & Atmospheric Research, New Zealand

## Abstract

Intertidal ecosystems have primarily been studied using field-based sampling; remote sensing offers the ability to collect data over large areas in a snapshot of time that could complement field-based sampling methods by extrapolating them into the wider spatial and temporal context. Conventional remote sensing tools (such as satellite and aircraft imaging) provide data at limited spatial and temporal resolutions and relatively high costs for small-scale environmental science and ecologically-focussed studies. In this paper, we describe a low-cost, kite-based imaging system and photogrammetric/mapping procedure that was developed for constructing high-resolution, three-dimensional, multi-spectral terrain models of intertidal rocky shores. The processing procedure uses automatic image feature detection and matching, structure-from-motion and photo-textured terrain surface reconstruction algorithms that require minimal human input and only a small number of ground control points and allow the use of cheap, consumer-grade digital cameras. The resulting maps combine imagery at visible and near-infrared wavelengths and topographic information at sub-centimeter resolutions over an intertidal shoreline 200 m long, thus enabling spatial properties of the intertidal environment to be determined across a hierarchy of spatial scales. Results of the system are presented for an intertidal rocky shore at Jervis Bay, New South Wales, Australia. Potential uses of this technique include mapping of plant (micro- and macro-algae) and animal (e.g. gastropods) assemblages at multiple spatial and temporal scales.

## Introduction

Plant and animal assemblages that live in intertidal regions such as rocky shores are part of a complex, dynamic ecosystem, the structure and functioning of which can vary across a cascade of spatial and temporal scales [1]. Intertidal ecosystems have primarily been studied using field-based sampling e.g. [2]–[5] at appropriate resolutions to capture the spatial variability at which assemblages occur. When studies cover a broad area of shoreline or occur at multiple sites, extensive field-based sampling requires a large amount of logistical effort and data is often not recorded in a contiguous manner (i.e. different parts of the shoreline are sampled at different times). Remote sensing is the ideal tool to collect contiguous data over large areas in a snapshot of time, however conventional remote sensing platforms (i.e. satellites and manned-aircraft) provide data at relatively coarse spatial and temporal resolution. Current state-of-the-art commercially available high-resolution satellite imagery can provide resolutions of 2.4 m per pixel for multi-spectral imagery and 0.6 m per pixel for panchromatic imagery, at a cost of $3000-5000US per imagery scene [6]. Manned aerial photography and airborne lidar provide higher resolutions (up to 0.3 m per sample point [7]), depending on flying height, and are typically more expensive, with targeted data collection costing in the order of tens of thousands of dollars per flight [8], [9]. The low-resolution and high costs of targeted data collection (i.e. at a specific time and place) limits the effectiveness of conventional remote sensing in small-scale environmental science and ecologically-focussed studies. Furthermore this data does not provide information on topographic variability at small scales (centimeters and meters), which is known to influence the distribution of assemblages of plant and animal species [10].

In this paper we develop data collection techniques and data processing algorithms for constructing ultra-high resolution (sub-centimeter) three-dimensional (3D) multi-spectral maps of intertidal rock platforms using a low-cost kite-based mapping system. The objective of our work is to develop a system that provides both topographic data and multi-spectral imagery over a broad area of the intertidal environment (hundreds of meters of shoreline) at appropriate spatial scales for ecologically-focussed studies. We describe a methodology for collecting images from two consumer-grade digital cameras, one that is a standard three-channel colour camera and the other a camera that has been converted to image in near-infrared (wavelengths greater than 720 nm). The cameras are carried on the flying line of single-line kite that is flown over the target site and used to collect multiple overlapping images of the terrain. The images are then processed in a procedure that automatically extracts and matches spatial features across multiple images and uses this information to incrementally reconstruct both a 3D map of the terrain and the position of the camera as each image was captured. Results are presented from a 200 m stretch of temperate intertidal rocky shoreline at Jervis Bay, New South Wales, Australia with an average topographic ground sampling distance of 2.5 cm and an imagery resolution of 5 mm per pixel. The resulting maps contain both topographic data and multi-spectral imagery. Topographic data allows for high-resolution measurements such as the vertical position on the shore with respect to tides and the slope and aspect with respect to the sun. The multi-spectral imagery (red, green, blue and near-infrared) is used to derive a Normalised Difference Vegetation Index (NDVI) that allows for the identification of intertidal species present during imaging and the potential to provide data on variables such as chlorophyll and algal biomass. The use of readily available and cheap consumer-grade equipment and the non-technically demanding process of collecting images in the field will hopefully allow for high-temporal resolution repeat sampling (i.e. in the order of days to weeks).

### Related Work

#### Intertidal Ecology and Remote Sensing

The distribution of assemblages of plant and animal species in rocky intertidal and soft sedimentary intertidal systems is known to be highly variable across a cascade of spatial scales ranging from cm to km [4], [11]–[16]. A complex interplay of bottom-up and top-down ecological processes are thought to regulate spatial distributions [17]. Remote sensing is able to provide the synoptic context in which spatial variations in assemblages can be quantified, yet the use of remote sensing has been limited by poor resolution.

Manned aircraft-based remote sensing has been used to study intertidal environments over large scales and low-spatial resolution. In [18], airborne hyperspectral imagery at a spatial resolution of 5 m was used to map the fractional coverage of macroalgae over a one kilometer-long rocky shore. In [19], airborne hyperspectral sensing was acquired at a 4 m resolution to measure sediment grain size and microphytobenthic biomass over several kilometres of an intertidal flat.

The advent of low-cost field spectroradiometers has resulted in a large body of work in field-based spectroscopy in recent years [3]. Spectroscopy in the visible and near-infrared bands has allowed for the measurement of indices relating to macrophyte cover [20] and microphytobenthic biomass [21] through the correlation of chlorophyll concentration and reflectance at different red and near-infrared bands. In [22] similar indices were derived for use with 3-band digital camera using red, green and near-infrared bands.

3D photogrammetry obtained from in-field, ground-based photography has been used to measure habitat structure and topographical complexity on rock intertidal shores. In [2] ground-based stereo-photos and photogrammetry techniques were used to measure different forms of structural complexity along repeated 30 cm transects to study the correlation to gastropod abundance in both rocky intertidal and mangrove habitats. In [5] similar field-based photogrammetry were used to measure topographic complexity along with surface temperature measurements to study the influence of habitat structure on body size and abundance of different invertebrates on an intertidal rocky shore.

#### Kite and Balloon Photography

Kites and balloons have been used for low-altitude aerial photography for decades, most prolifically in archaeology (see [23] for a good review) as a low-cost means of recording cultural heritage or providing a visual record of excavation activities. Balloons have typically been used when windspeeds in an area are low and kites when windspeeds are higher, providing complimentary use in a range of environmental conditions. These platforms are typically constructed from low-cost materials, are safe due to their low-weight and stability and easy to deploy in a range of conditions promoting their use in a wide range of applications such as boat-launched coral aerial photography [24], counting Antarctic penguin numbers [25] and monitoring in a humanitarian emergency [26]. In [27] the authors describe the use kite aerial photography in environmental site investigation and [28] discusses the complexities of kite aerial photography using near-infrared film in the study of vegetation and soil properties. In [29], the authors develop a kite aerial photography platform that includes real-time pan/tilt control and image stabilisation. Previous work in these applications has often focussed on taking either a single or a small number of photographs.

More recently kite aerial colour photography has been used to map and classify different vegetation types in the alpine zone using both supervised classification and unsupervised clustering of the three-band colour imagery [30]. The work in [31] and [32] used kite aerial photography to produce digital elevation models from a small collection of photographs producing topographic maps with pixel sizes of 4 cm and 5–7.5 cm over an area of 100-by-100 m and 50-by-50 m respectively. In [32] topographic maps were used to track the progress of gully erosion over a period of 2–4 years.

In relation to studies in the intertidal zone, the work in [33] used a helium blimp to take photographic stereo pairs of a section of rocky intertidal shore using both colour and near-infrared film. The photographs were geo-referenced to an 18-by-18 m plot at a resolution of 2 cm per pixel.

#### Unmanned Aerial Vehicles (UAVs)

Unmanned Aerial Vehicles (UAVs) [34] are a relatively recent technology that have been used to produce high resolution maps owing to their ability for low-altitude flight. In [35], the authors demonstrated the use of UAVs for collecting images over rangelands with a spatial resolution of 5 cm per pixel. In the work of [36], a fixed-wing UAV was used to produce geo-referenced imagery maps with a resolution of 3.5 cm per pixel over an area of 4000-by-600 m in a weed monitoring application. Hovering UAVs (such as the Mikrokopter (http://mikrokopter.org)) have been demonstrated as a promising platform for low-altitude sensing for even higher resolution imaging. A hovering UAV was used to produce maps of Antarctic moss beds with a resolution of 1 cm per pixel over an area 100-by-40 m in [37]. In [38] the authors used hovering UAV imagery to produce 3D maps of a coastal cliff, producing pointclouds with a spatial resolution of 1–3 cm and an accuracy of 25–40 mm. UAVs are promising platforms for gathering high-resolution remotely sensed data; although the costs and technical skills required to operate these platforms are becoming lower over time, they are still relatively high, particularly when considering their use in small-scale ecological studies. Additionally, the current generation of rotary-wing UAVs are typically limited by low endurance (approx. 15–20 minutes) and are susceptible to failure in high-wind conditions, typically encountered in coastal regions.

#### Recent Developments in Photogrammetry and Structure-from-Motion

The process of measuring spatial properties from photographs or images is referred to as ‘photogrammetry’ and when large numbers of images are used, typically to reconstruct the three-dimensional spatial structure of an imaged scene, this process is referred to as ‘structure-from-motion’ [39]. Recent developments in structure-from-motion [40], [41] have focussed on building 3D models of buildings from large collections of un-ordered, un-calibrated images. These methods utilise multi-core and parallel processing algorithms for efficiently combining images with relatively little additional information such as from Global Positioning Systems (GPS) or ground control points.

### Overview of the contributions of our work in contrast to related work

In this paper we combine aspects of photogrammetry, multi-spectral remote sensing and low-cost image collection using a kite-based platform for building high-resolution imagery and terrain models in the intertidal zone. In contrast to previous work in kite aerial photography, our method fuses information from hundreds, and potentially thousands of overlapping monocular images using modern photogrammetry and bundle adjustment techniques [40], [41]. This enables coverage over much larger areas and with higher spatial resolution. The photogrammetry techniques used employ algorithms for automatic calibration of the intrinsic and extrinsic parameters of the camera (i.e. focal length, lens distortion etc.) using the images themselves, allowing for the use of un-calibrated, low-cost consumer-grade cameras. The techniques also construct full, scale-less 3D models prior to the use of ground control points, providing flexibility in the way in which models are geo-referenced into a global coordinate system. Furthermore, we demonstrate a low-cost method for producing spatially-registered *multi-spectral* imagery from multiple consumer-grade cameras and validate the utility of this information for distinguishing macroalgae in the intertidal environment through the use of the NDVI. Compared to past work on intertidal mapping using traditional remote sensing platforms or field-based sampling, our approach provides data with an unprecedented level of resolution over a broad-scale environment. The data collection procedure is fast and easy to perform without technical experts in remote sensing or UAV operations.

## Materials and Methods

### Study Site

**Figure 1:**
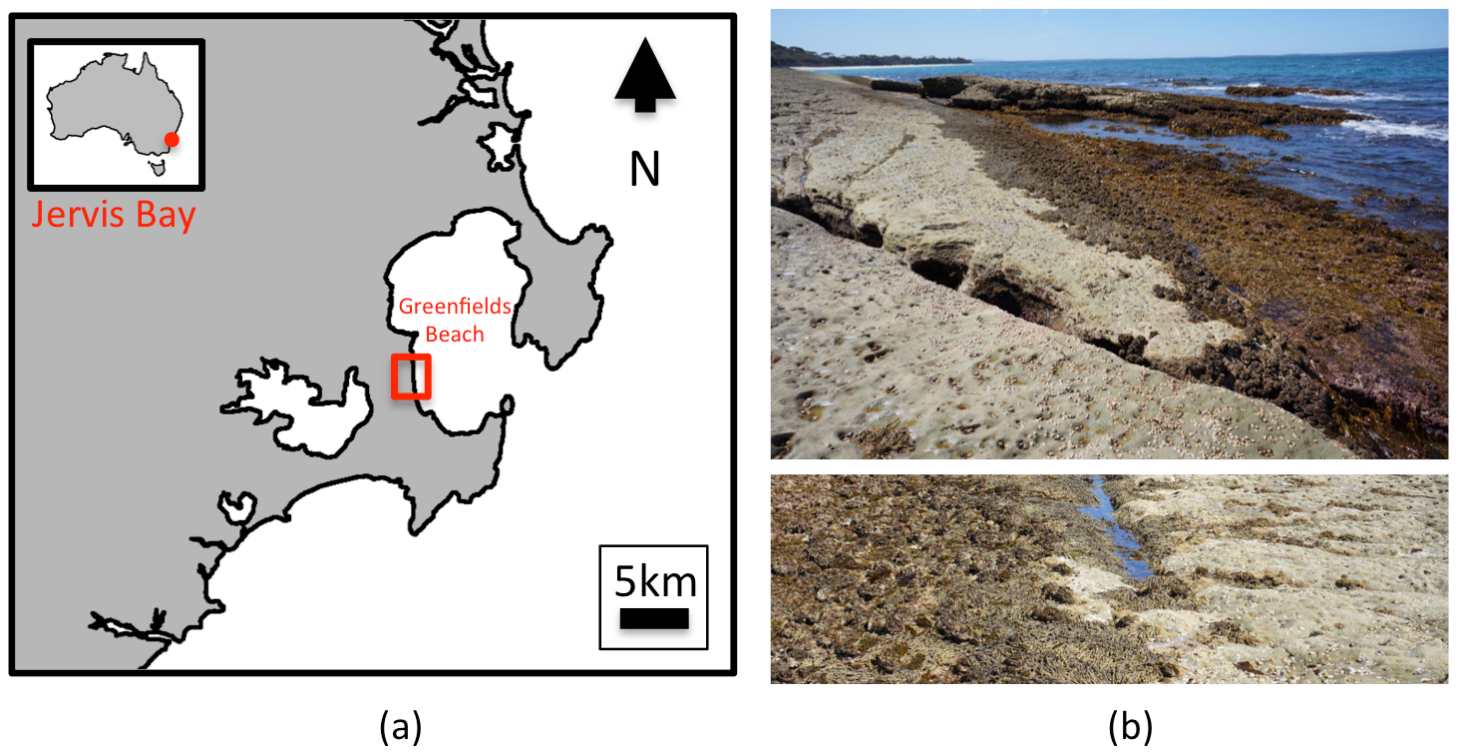
Study Site. (a) Intertidal rocky shore at Greenfields Beach (study site shown in red box) at Jervis Bay, New South Wales, Australia, (b) ground-based photography at the study site taken at low-tide. Google maps imagery of the site is available at: http://maps.google.com.au/?ll=-35.085668,150.693294&spn=0.005759,0.0109&t=h&z=17.

Experiments were performed over an intertidal rocky shore north of Greenfields beach (34.000°S, 151.249°E) along the western edge of Jervis Bay, New South Wales, Australia (see Figure 1). The site lies within a national park aquatic reserve and is host to various intertidal species and is largely populated by red, green and brown macro-algae such as *Homosira*, *Corallina*, *Ulva* and *Ralfsia* along the low shore. Various gastropods and tunicates (cunjevoi) were also present. Data collection was performed over a single, cloudless day during October 2012 at approximately 1:50 pm to coincide with a low tide of 0.16 m above the datum. All field studies involved only non-contact, non-invasive means of sampling (i.e. collection of images and field spectroscopy) and therefore did not require any specific permissions or ethics approvals.

### Kite Aerial Photography Equipment

**Figure 2:**
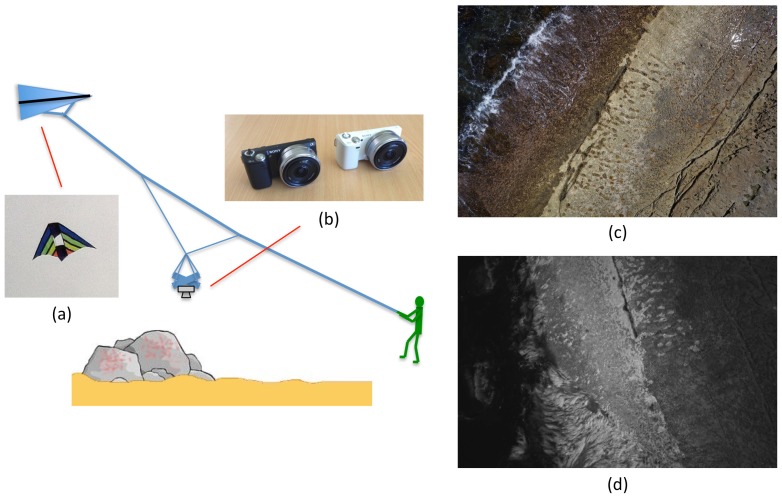
Kite-based image acquisition. (a) 2.7 m wingspan conynes-delta kite using to lift (b) a Picavet suspension rig that was used to attach each of the two downwards-facing Sony NEX-5 digital cameras (one colour and one near-infrared). The operator walks the kite across the intertidal zone collecting multiple, overlapping photographs. Examples of aerial images collected from an altitude of approximately 15 m are shown in colour (c) and near-infrared (d).

A kite aerial photography system (see Figure 2) was built that used a 2.7 m wingspan conynes-delta kite to lift a fixed, downwards-looking rig holding a consumer-grade digital camera. The conynes-delta kite was chosen for its stability and lifting capacity in a wide range of wind conditions. The camera was suspended from a Picavet rig that attached to the line of the kite approximately 10 m lower than the kite to minimise the impact of wind gust-induced motion of the camera. The Picavet provided mechanical levelling of the camera during changes in the flying angle of the kite. Two different versions of a consumer-grade digital camera (the Sony NEX-5 with a 16 mm pancake lens and a 16 MPix resolution) were used - a standard three-colour camera and one that had been converted for imaging in near-infrared wavelengths. A commercial service (LifePixel) was used to remove the internal near-infrared cut filter (installed in the majority of consumer digital camera to remove near-infrared light from images) and replace it with a high-pass colour cut filter that allowed transmission of wavelengths above 720 nm. The camera was chosen as a trade-off between the image quality achieved from a full-frame sensor digital single-lens reflex camera and the light-weight of a small-sensored compact digital camera.

**Figure 3:**
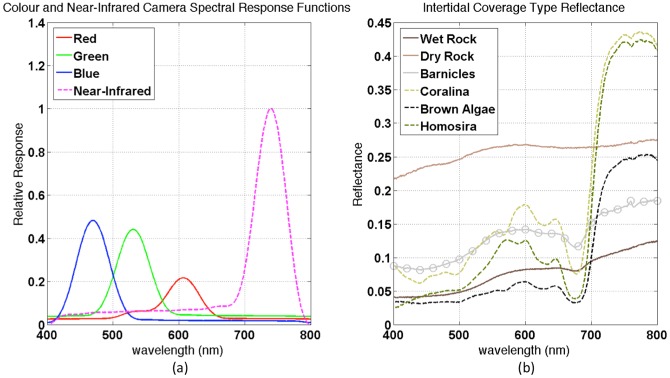
Spectral calibration data. (a) Spectral response functions for the colour and near-infrared converted cameras: the red, green and blue channels correspond to the three channels of the colour camera whereas the near-infrared curve corresponds to the red channel of the near-infrared converted camera, which was found to have the highest response of each of the channels for this camera. (b) Reflectance spectra for key surface coverage types in the intertidal zone measured using a handheld spectroradiometer. The reflectance spectra were used in conjunction with the camera spectral response functions to validate the measured colour of objects in the kite-based imagery.

The spectral response functions of both cameras were determined using the procedure described in [42], [43] to provide precise information on the spectral sensitivities of the raw imagery from the red, green and blue channels of the colour camera and the near-infrared sensitivity of the converted camera (data was taken only from the red bayer channel of the images for this camera). Images of a Macbeth colour chart were captured using the raw mode of each camera under cloudless sunlight and measurements of the spectral response of each Macbeth colour panel were taken using a handheld spectroradiometer (Ocean Optics STS-VIS with an effective range from 400–800 nm), from which the response curves were estimated. Although the panels of the Macbeth colour chart are designed for use in colour calibration in the visible spectrum, it was found that the panels exhibited variations in reflectance at wavelengths around 720 nm and above, and were therefore also effective for calibrating images captured from the near-infrared converted camera. Figure 3 (a) illustrates the estimated spectral response curves for the cameras.

The red, green and blue channels of the colour camera displayed peaks at 470 nm, 530 nm and 606 nm respectively and the red channel of the near-infrared camera (which was found to have the best response of the three colour channels of this camera) had a peak response at 740 nm (shown in Table 1).

**Table 1:** Peak response values of the colour and near-infrared converted cameras.

	Red	Green	Blue	Near-infrared
Peak Response	470 nm	530 nm	606 nm	740 nm

The red, green and blue values correspond to the three channels of the colour camera whereas the near-infrared value corresponds to the red channel of the near-infrared converted camera.

### Process Overview

**Figure 4:**
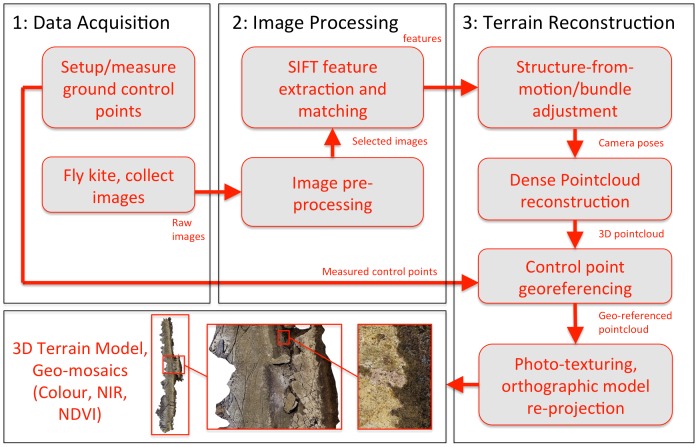
Overview of the kite-based mapping process. During data acquisition, ground control points are placed in the environment and images collected over the the terrain using the kite and camera. After data acquisition, images are processed to extract and match features across multiple overlapping images. These features are used to reconstruct the poses from which images were captured and a 3D pointcloud of the terrain using a structure-from-motion algorithm. The pointcloud is geo-referenced using the ground control points and a photo-texturing process is used to create 3D topographic maps and high-resolution geo-mosaics.

Figure 4 presents a flowchart illustrating the entire kite-based mapping process performed in the study. The process began by placing ground control points (visible markers) across the terrain and using the kite-based platform for collecting a series of overlapping images of the terrain taken from the air. After the images were acquired, they were processed using an automated procedure to produce 3D terrain models and geo-referenced photo-mosaics of the area covered by the images. The automated procedure extracted and matched feature points that were used by a structure-from-motion algorithm to reconstruct the poses from which the images were taken and a 3D point cloud of the imaged terrain. After geo-referencing this pointcloud using the measured ground control points, a photo-texturing algorithm was used to create the final map products. Table 2 provides an overview of the different software packages used at various stages in the data processing procedure. The following subsections describe these steps in detail.

**Table 2:** Overview of software implementations used within the processing procedure.

Task	Software Used
Raw image conversion	DCRAW (http://www.cybercom.net/~dcoffin/dcraw/)
SIFT Feature Extraction	vlfeat (http://www.vlfeat.org/)
Feature Matching	Customised multi-core implementation using [46] and ANN (http://www.cs.umd.edu/~mount/ANN/)
Structure-from-motion, Bundle Adjustment	Bundler (http://www.cs.cornell.edu/~snavely/bundler/)
Multi-view Stereo Triangulation	PMVS2 (http://www.di.ens.fr/pmvs/)
GCP Geo-registration	Customised implementation using [49]
Photo-textured Terrain Modelling and Mosaicing	Custom implementation, software and code available: (Structured: https://github.com/mattjr/structured)

### Spatial and Spectral Ground Control Points

In order to geo-reference the 3D landscape models and maps reconstructed from the collected image data, flat 20-by-20 cm checkerboard-patterned panels were placed on dry rock surfaces in the environment before flight to act as ground control points. The position of each panel was measured using a GPS receiver. Because of the focus on low-cost equipment used in this study, ground control point panels were measured using a consumer-grade handheld GPS receiver that provided global position coordinates to an accuracy of ±2 m (rather than using a more expensive survey grade, differentially corrected GPS device that could potentially provide cm-level global accuracy). In order to supplement the low-accuracy GPS measurements, additional spatial constraints were measured between the ground control points; the points were placed in triads, where each triad was arranged into an equilateral triangle pattern with an hand-measured edge length of 2 m (using a tape measure). A total of nine ground control points were placed into the environment. More details on how the control points were used are discussed in the subsection on ground control point geo-registration below.

In addition to the ground control points, ground control reference spectra were measured for various surfaces in the intertidal zone using the handheld spectroradiometer. The positions of the spectrally-measured points relative to the ground control point targets were measured so that they could be later identified in the reconstructed maps and used to validate the accuracy of the colour and near-infrared data in the maps. For each measured spectra an estimate of the expected red, green, blue and near-infrared responses as seen by the cameras was produced by multiplying the measured spectra with the estimated spectral response curves of the camera (see Figure 3 (a)) and summating over wavelength for each channel. The red and near-infrared channels were then used to produce a macrobenthos index that was compared to the index computed using the actual red and near-infrared responses measured from the camera. Figure 3 (b) illustrates examples of the reflectance spectral curves for dominant coverage types found at the Jervis Bay field site.

### Image Acquisition

During data collection, the kite was used to hoist the camera rig holding one camera at a time over the area of interest and the camera programmed to capture images at a frequency of approximately one shot per second using the continuous shoot mode of the camera. The aperture, exposure time and ISO were tuned by hand for each camera to the average light conditions on the day and kept constant for every image captured by a given camera. The actual values used were different for each camera (owing to the differences in the transmission properties of the internal filters of each camera). A white-balance procedure using a Spectralon target was used to balance the intensity of image data between cameras; this procedure is discussed more detail in the section below on NDVI mosaics. The kite could be flown at a variety of altitudes between approximately 10–100 m (limited by the length of the kite line) based on desired area coverage and ground spatial resolution. The desired height was achieved using distance markers on the line and by approximating the flight angle of the kite. The kite was then slowly walked across a 200-by-30 m section of rocky shoreline and images were captured continuously using the raw mode of the camera at an altitude of approximately 15–20 m. The kite was walked in a zig-zag fashion along the shoreline rather than in a straight line in order to gather images from various perspectives with respect to the terrain. Capturing images from various perspectives was important for the functioning of the structure-from-motion algorithms discussed below, allowing for camera poses and 3D feature information to be estimated. The time taken to acquire images across the platform was approximately six minutes, after which the cameras were swapped and the process repeated to collect images in both colour and near-infrared. It was originally planned that both cameras would be flown simultaneously, however light wind conditions on the day only allowed for one camera to be flown at a time.

Figures 2 (c) and (d) illustrate example images captured by the system at an altitude of approximately 15 m from the ground, with a coverage footprint of approximately 22-by-15 m and a pixel size of approximately 4.5 mm.

### Image Processing, Feature Extraction and Matching

After data collection, images were copied from each of the cameras to a desktop computer for processing. Prior to processing, images that were affected by motion blur during wind gusts or large occlusions of the terrain (for example images of people moving in the scene) were removed manually. Images were white balanced, to aid in feature extraction contrast and to provide imagery mosaics that could be easily interpreted by end-users. A total of 295 colour images and 251 near-infrared images were used. Scale-Invariant Feature Transform (SIFT) features were extracted in each image (using the implementation in [44]) and matched across all image pairs (including colour to colour, colour to near-infrared and near-infrared to near-infrared images) using a kd-tree [45] of the features present in each image. SIFT features correspond to distinctive points in the texture of surfaces captured in images and were well suited for use in the rocky intertidal environment.

Although there existed types of terrain that had a different appearance in the colour and near-infrared images, enough similarity in the properties used in the SIFT feature descriptor was present to match and register SIFT features across these two different image formats. Table 3 shows the number of matching image pairs and average feature matches per image for colour-to-colour, colour-to-near-infrared and near-infrared-to-near-infrared image pairs. The average number of features per image matched between the colour and near-infrared images was lower than colour-to-colour or near-infrared-to-near-infrared pairs. Enough matches were found to provides a means to registering different types of image data into a common reference frame during the remainder of the processing procedure.

**Table 3:** Comparison of the number of matching image pairs and average feature matches per image for colour-to-colour, colour-to-near-infrared and near-infrared-to-near-infrared image pairs.

	colour to colour	colour to near-infrared	near-infrared to near-infrared
Number of matching image pairs	4454	3063	2630
Average number of matching features per pair	854.6	188.2	1106.2

**Figure 5:**
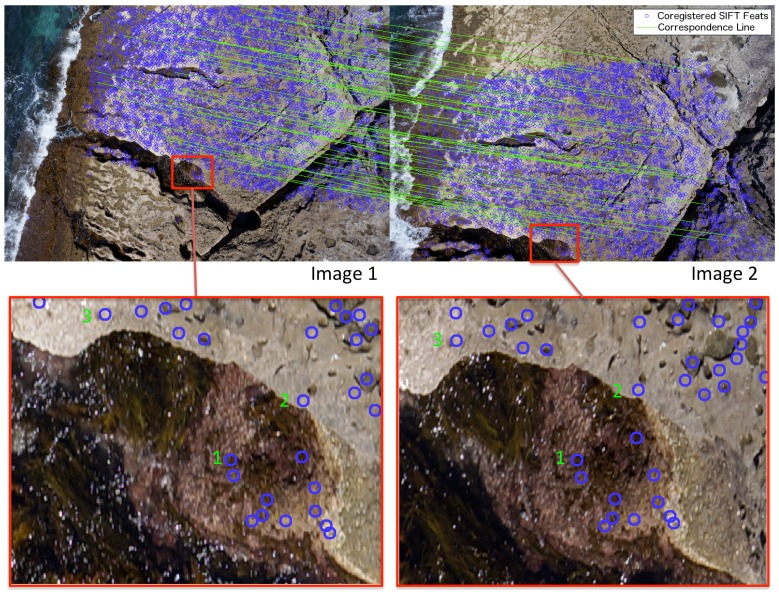
Example of extracted and matched SIFT image feature data. Shown are two overlapping images (image 1 and image 2) with annotated positions of SIFT features that have been matched between the two images (blue) and lines displaying the computed correspondence between points (green) (only every hundredth correspondence shown for clarity). Also shown are detailed sections of each image illustrating the feature points.

Robust detection of incorrect feature matches was performed using epipolar constraints between images [46]. Multi-core software implementations of these methods were developed in order to process images in parallel, speeding up the total time taken for processing. Figure 5 illustrates an example of the extracted and matched feature points between two images.

### Structure-from-motion and Pointcloud Reconstruction

A structure-from-motion/bundle adjustment software package [47] was then used to incrementally construct a 3D point feature map corresponding to the matched image feature points while simultaneously estimating camera poses and the intrinsic and extrinsic parameters for the camera. The method used was able to build a complete, scale-less 3D reconstruction (but with unknown global orientation and position) by using only the image data. The method employed a simplified model of the camera extrinsics (including focal length and lens distortion), the parameters of which were estimated from the image data and matched features themselves (as opposed to use of a pre-calibrated or metric camera). A multi-view stereo reconstruction algorithm [48] based on the correlation score of dense patches in the overlapping images was then used to produce a dense 3D point-cloud corresponding to a higher spatial resolution than by using SIFT features alone. This algorithm used the relative camera poses estimated during bundle adjustment to triangulate dense image features and robustly remove outliers from the terrain point cloud. The resulting 3D pointcloud had a spatial density that depended on the level of texture in the environment and was usually within a small factor of the image pixel size (i.e. approximately one 3D feature for every 5-by-5 pixel patch on average).

### Ground Control Point Geo-registration

The ground control points that had been placed into the environment were identified in the pointcloud reconstruction and used in two ways to recover the scale, position and orientation of the final reconstruction. Firstly, the hand-measured triangle edge lengths were used to compute the absolute scale of the 3D reconstruction (i.e. the size of the model in the world) by comparing to the edge lengths of the triads of control points identified in the pointcloud reconstruction. Secondly, the GPS measured coordinates were used via Horn's method [49] to compute a transformation consisting of a translation and orientation that moved the scaled 3D model from arbitrary reconstruction coordinates into a geo-referenced coordinate system (i.e. latitude, longitude and altitude). Because of the low global accuracy of the GPS measured coordinates, the ground control points were not directly integrated into the structure-from-motion process.

The two different steps allowed for different information (with different levels of accuracy) to be used to compute the scale of the model and the position/orientation of the model. The scale of the landscape model was recovered accurately (owing the precision of the hand measured edge lengths) whereas the global position of the model (i.e. the absolution latitude, longitude and altitude of the model) was recovered with less accuracy (owing to the precision of the GPS measurements themselves). This distinction in the types of accuracy is important; often the local accuracy of the map (i.e. the error in the relative position of two objects in different parts of the map) is important, whereas the global accuracy of the map (i.e. the error in the absolution latitude, longitude and altitude of a given point) is of less importance. The same methods applied in our work could be used with higher-accuracy GPS ground control point measurements to achieve both high local and global accuracy in the map, but at the cost of requiring more expensive equipment.

In order to improve on the vertical accuracy of the model (owing to the poor vertical resolution of the GPS), the vertical elevation of the reconstruction was adjusted by manually extracting an outline of the water line visible in the point cloud and setting this to the known tidal datum height around the time the images were captured.

### Photo-textured Terrain Model and Mosaicing

A triangulated terrain surface model was constructed from the 3D geo-referenced pointcloud using Delaunay triangulation [50]. For each face of the surface, the estimated camera poses were used to compute which images had seen the face both from the colour and near-infrared images. The images were grouped into two lists, one composed of colour images and one of near-infrared images. For each list, images were ranked based on the distance between the centerpoint of the face in the environment and the camera position from which the image was taken (and thus image resolution at this point). The best four images (those with minimum distance) from each list were then assigned to the face and the 3D coordinates of the face were projected into the 2D coordinates of each image. Using the projected coordinates, a colour value was assigned to each pixel in the final texture using a weighted averaging of these four source images according to the algorithm in [51]. The process was repeated for every face in the model to produce two sets of photographic textures, one corresponding to colour imagery and one corresponding to near-infrared imagery on the surface. The view selection and band-limited-blending of [51] allowed for only the best images of a given surface to be used in the final model, providing redundancy against images taken from poor angles.

The resulting 3D photo-textured model was visualized using a level-of-detail rendering system [51] to capture sub-centimeter details over the entire span of the map. Additionally orthographic projections of the model (imaged from directly above) were re-rendered in separate colour and near-infrared bands to produce 2D photomosaics of the entire area that were exported to a geotiff format.

### Spatial and Normalised Difference Vegetation Index (NDVI) Mosaics

In addition to imagery mosaics, maps of various spatial properties including terrain elevation, slope and aspect were generated from the 3D terrain model. Slope and aspect were computed for each triangular face in the terrain model based on the normal vector of the face 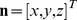 (where 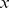, 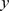 and 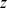 are the north, east and down components of the normal) using Equations 1 and 2:
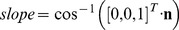
(1)

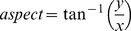
(2)


The colour and near-infrared imagery layers were also used to compute the Normalised Difference Vegetation Index (NDVI) at each spatial point using a combination of the red and near-infrared (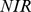) channels of the imagery:
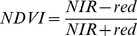
(3)Data was taken from the raw imagery collected from each camera after a custom white balance procedure was applied to each channel. The procedure used images captured from each camera of a white Spectralon target under the same lighting conditions in which the imagery was collected using the kite. Using the measurements of the Spectralon target, for each camera a white balance gain was applied to each of the red and near-infrared channels that normalised the outputs to the ratio between the exposure times of each camera. The resulting white-balanced channels were then used to calculate the NDVI using Equation 3. This version of NDVI is closely related to the microphytobenthos index described in [21] that used precise ratios of red and near-infrared reflectance from a spectroradiometer at wavelengths of 635 and 750 nm and was found to have a linear relationship to surface algae chlorophyll concentration. In our work, the red and near-infrared channels provided by the imagery had slightly different peak response values (606 and 740 nm respectively, see Table 1) and also collected light over a wider band of wavelengths (i.e. Figure 3 (a)).

## Results and Discussion

### Spatial Mapping Results

**Figure 6:**
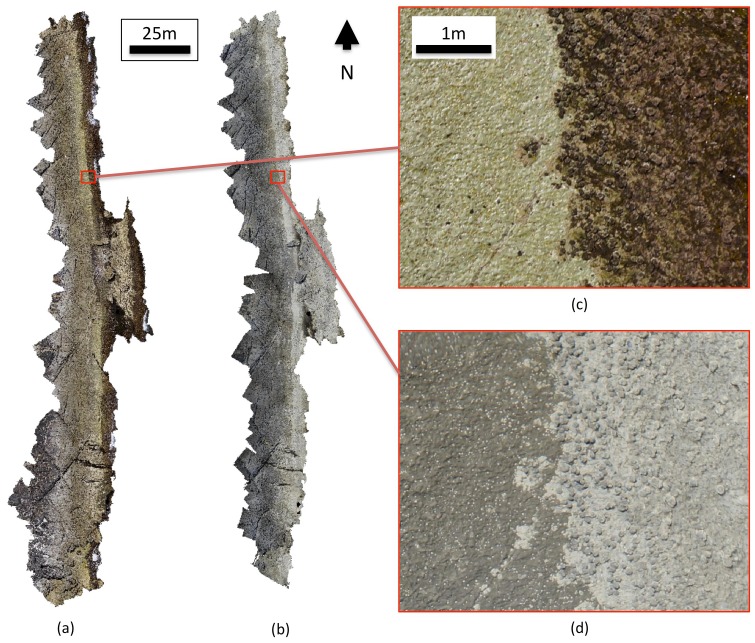
Colour and near-infrared photomosaic layers of the intertidal rocky shore reconstruction at Greenfields Beach. (a) Colour mosaic and (b) near-infrared mosaic for the whole shoreline. (c) and (d) show a detailed section of the colour and near-infrared mosaics illustrating the different scales achieved across the entire map.

Figure 6 illustrates the final photo-mosaic of the intertidal rocky shore for the colour and near-infrared photomosaic layers. The coverage achieved in each map corresponds to the locations where points on the ground were observed in at least two different camera images (and thus could be reconstructed using the structure-from-motion techniques discussed above). Coverage in the colour and near-infrared mosaics was slightly different owing to the fact that some parts of the terrain were observed only by a single type of camera. The final spatial resolution of the mosaic was 5 mm per pixel on average, with slight variations owing to variations in the flying height of the kite and the perspective from which images were captured. The detailed sections (shown in Figure 6 (c) and (d)) illustrate a division along the shoreline between the upper tidal zone, populated largely by bare rock with various grazing gastropods such as limpets and other sessile organisms such as barnacles and the mid-tidal zone populated largely by a patchwork of macroalgae and cunjevoi.

**Figure 7:**
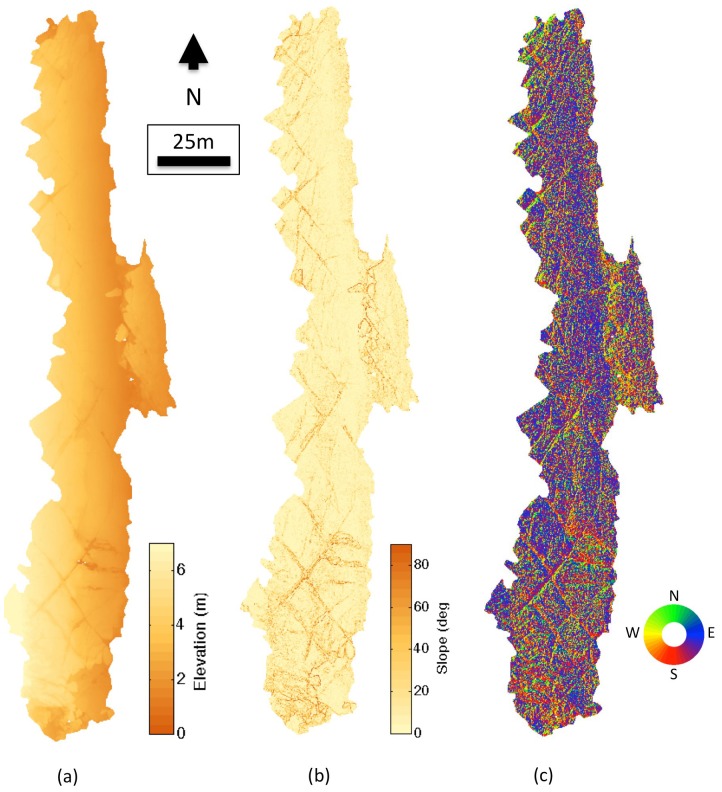
Elevation, slope and aspect data derived from the 3D topographic reconstruction. (a) Elevation above maximum low tide (b) slope of the terrain and (c) aspect of the terrain.

**Figure 8:**
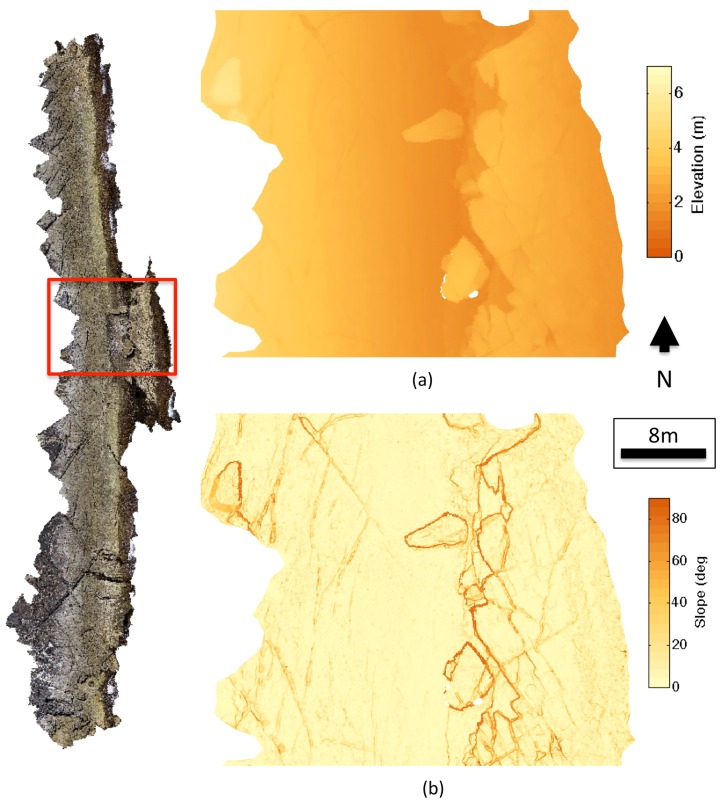
Detailed views of elevation and slope data derived from the 3D topographic reconstruction. (a) Elevation above maximum low tide and (b) slope of the terrain.

**Figure 9:**
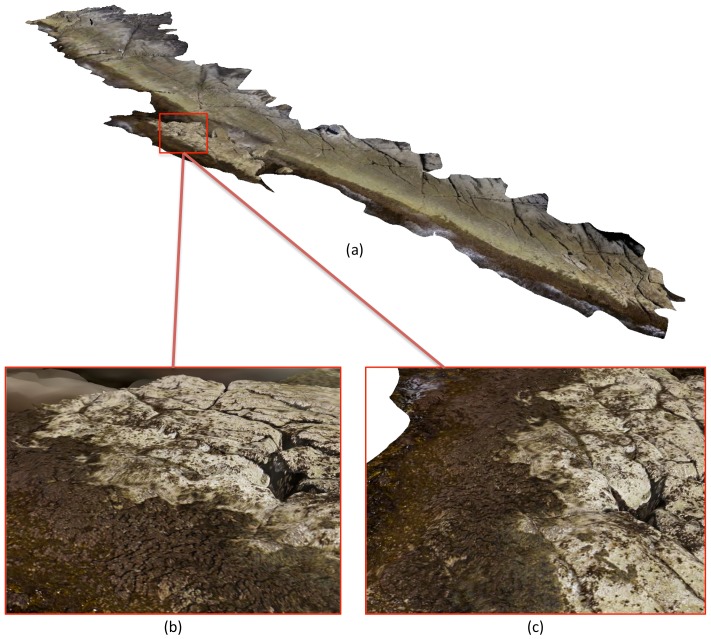
3D photo-textured rocky shore reconstruction. (a) 3D oblique view of the shoreline, (b) and (c) Detailed oblique views of rock platform section from different viewing angles. The level-of-detail visualisation system allows for different model scales to be visualised in a single, continuous terrain model.

Figures 7 and 8 show the elevation, slope and aspect maps derived from the 3D terrain surface map. The final 3D terrain map displayed an average 3D topographic ground sampling distance of 2.5 cm. The fine-scale structure of rock pools and crevasses is visible from the maps. Figure 9 illustrates a visualisation of the final photo-textured 3D model with various views from large-scale to fine-scale. The level-of-detail visualisation system allowed for different model scales to be visualised in a single, continuous terrain model. The highest level of detail information provided detailed structural information on individual rock crevasses with imagery information indicating the presence of different macroalgae coverage and assemblages of barnacles and limpets.

### Validation of Spatial Accuracy

The residual error between the measured ground control point locations and the computed ground control point locations produced in the final maps was used as a proxy for the estimated spatial accuracy of the 3D reconstruction and photomosaics. Two different types of spatial accuracy were assessed in the maps: global accuracy (i.e. absolute position of the entire map in geo-referenced coordinates) and local/relative accuracy (i.e. the accuracy in the reconstructed relative position of two different points on the map). The expected global accuracy was assessed using the residual errors between the GPS-measured ground control point locations and the identified ground control point locations produced in the final maps. The local/relative accuracy was assessed using the residual error between the hand-measured ground control point triangle edge lengths and the edge lengths extracted from the 3D reconstructed maps. The resulting averages of the residual errors are shown in Table 4. In both cases, the residual error was in the order of the errors associated with the external measurements themselves (i.e. GPS-based residual errors of 1.016 m compared to the accuracy of the handheld GPS, which was reported by the GPS receiver as being ±2 m and the triangle edge length residual of 0.039 m compared to the expected precision of the hand made tape measurements, which was on the order of a few centimeters).

**Table 4:** Measures of both global and local-scale (fine-scale) spatial map errors from Ground Control Point (GCP) residual errors.

Average GCP Residual (Global-scale Error)	Average GCP Triangle Edge-length Residual (Local-scale Error)
1.016 m	0.039 m

In comparison to other studies using kite aerial photography, position errors of 0.02–0.14 cm were reported in [32], capturing images at a height of approximately 15 m and errors of 0.02–0.06 m in [33] capturing images at a height of approximately 50 m. In both of these studies, ground control points were positioned using a total-station survey and survey-grade GPS with an accuracy of 1 cm. Errors of 0.025–0.04 m were reported in [38] in aero-triangulated points computed using images from a UAV flying at approximately 50 m altitude. In this study, ground control points were measured using a Real Time Kinematic (RTK) Differential GPS system with a reported accuracy of approximately 1–4 cm. All of these studies report accuracies with respect to a geo-referenced coordinate system (i.e. global accuracies). The local/relative accuracy results reported in our work are comparable to the global accuracies of other studies, however the global accuracy of our approach is much lower (i.e. approximately 1 m compared to cm-level accuracy). This is a fundamental limitation of using low-grade (and thus inexpensive) GPS for surveying ground control points. The methods used in this paper are still applicable when survey-grade GPS measurements of ground control points are available; further analysis of local vs. global accuracy when using these type of measurements is left to future work.

### Normalised Difference Vegetation Index (NDVI) Mapping Results

**Figure 10:**
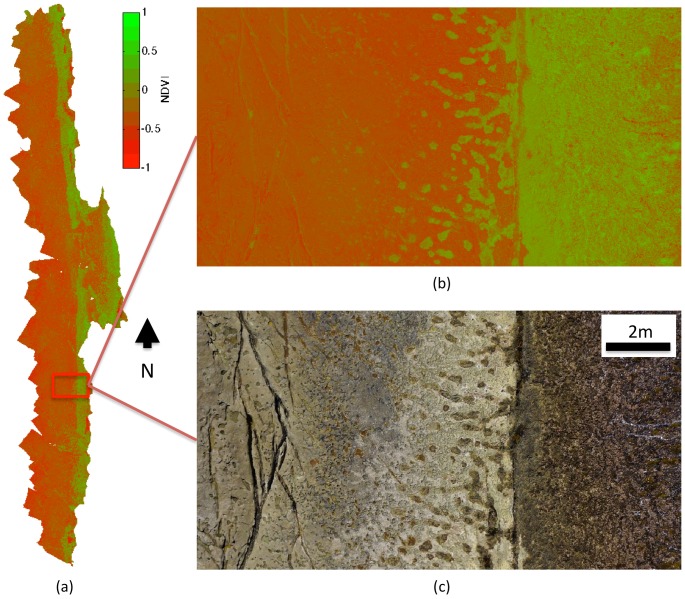
Normalised Difference Vegetation Index (NDVI) maps derived from raw colour and near-infrared imagery. (a) NDVI map for entire shoreline, (b) detailed view of NDVI and (c) corresponding colour imagery of detailed section.

Figure 10 shows the NDVI photomosaic layer computed from the custom white-balanced colour and near-infrared imagery using Equation 3. A comparison between the NDVI layer and the colour imagery layer (seen in Figures 10 (b) and (c)) of the shoreline illustrates the division between largely dry rock and an exposed mat of macroalgae and tunicates (cunjevoi). The presence of live micro- and macroalgae have been shown to correspond to NDVI values of 0.3 and 0.7 [21], bare ground and rock corresponding to values of approximately zero and water corresponding to values approaching −1.0. The NDVI mosaic allows for the inference of fine-scale spatial patterns of macro-algae to be identified from the imagery, for example within rock pools and crevasses along the upper intertidal zone.

### Validation of Spectral Accuracy

The ability of the reconstruction algorithms and consumer-grade camera images to reproduce accurate colour and NDVI in the mosaic maps was assessed by comparing the reconstructed textures in the maps to the ground control reference spectra that were collected by handheld spectroradiometer. The measured spectra were multiplied through the computed spectral response function of each of the red, green, blue and near-infrared camera channels and integrated to produce and expected red, green, blue and near-infrared responses, from which a predicted NDVI value was computed for each of the dominant intertidal coverage types. These values were then compared to the measured NDVI mosaics by examining 167 manually extracted 15-by-15 pixel patches corresponding to the dominant coverage types measured in Figure 3 (b).

**Figure 11:**
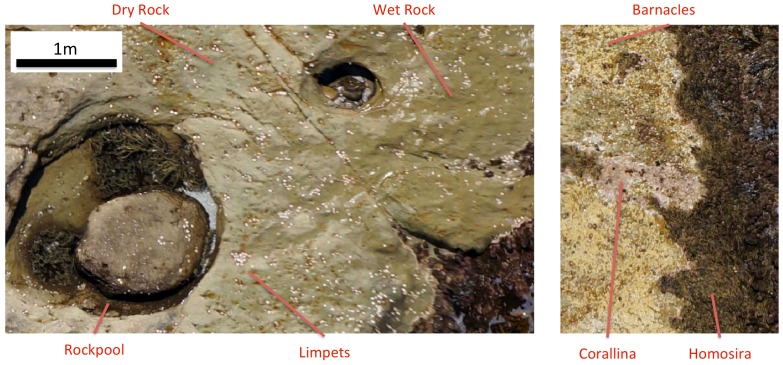
Example mosaic imagery highlighting some of the dominant surface types compared in this study.

**Figure 12:**
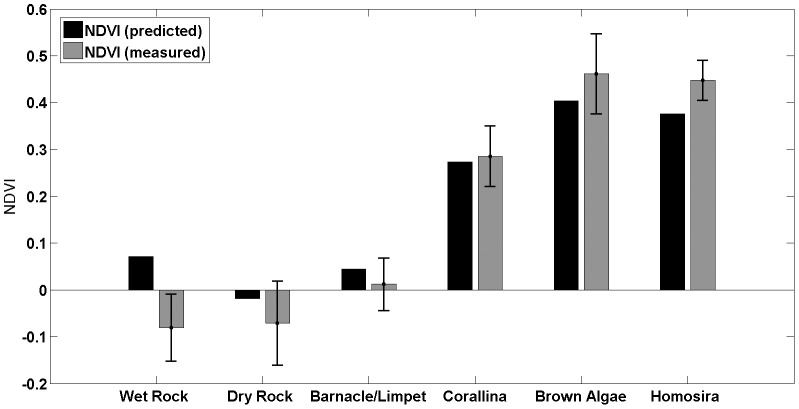
Comparison of predicted and measured Normalised Difference Vegetation Index (NDVI) values for different intertidal zone coverage types. Predicted NDVI values were derived using knowledge of coverage type reflectance spectra and camera spectral response functions (see Figure 3). Measured NDVI values correspond to manually extracted image patches taken from the reconstructed NDVI mosaic maps (error bars show the standard deviation from multiple sample points).

Figure 11 illustrates detailed views of the colour mosaic imagery showing examples of some of the dominant surface coverage types that were used in the comparison. Figure 12 illustrates the comparison for six different coverage types with error bars illustrating the variation in the manually extracted measurements. Overall there was a good correspondence between the measured and predicted NDVI values, with a large and obvious division between the macroalgae coverage types (with high NDVI) and the non-algal dominated surfaces (with NDVI close to zero). The ‘wet rock’ coverage type, displayed a difference between the predicted and measured NDVI outside of the error bars of the different measured samples. This was thought to be due to variations in the microalgal coverage on different wet rock surfaces in the intertidal zone that were not observable by eye during field sample collection and resulted in inaccuracies in the collected data.

### Online Data Availability

Results from the paper including mosaic geotiff files are available for download from: http://www-personal.acfr.usyd.edu.au/m.bryson/bryson_etal_2013.html.

## Discussion

### Potential Uses in Intertidal Ecology

The results presented here demonstrate the ability of our technique for collecting relevant spatial variables (i.e. elevation, slope, aspect) and spectral variables (such as NDVI) at fine spatial scales and over a broad area in the intertidal environment. The spatial variables are potentially useful for computing water emersion times, habitat structural complexity and seasonal sun exposure at different positions along the shoreline, while spectral indices such as NDVI are potentially useful for evaluating algal biomass. Similar variables have been used as part of studies into body size and abundance of invertebrates on rocky shores [2], [5], variations in microalgal biomass versus surface light availability on soft intertidal sediments [52], spatial distributions of intertidal macrobenthos versus food availability and sediment size [19] and for quantitating fractional macroalgae coverage [18]. Our low-cost method allows for collections of relevant spatial and spectral data across a cascade of fine-spatial scales, in a non-destructive manner, while preserving a permanent electronic record that can be used for comparison against repeated surveys, and thus could be an extremely useful tool within these type of studies.

Traditional remote sensing methods or field-based spectroscopy typically utilise high-spectral resolution measurements to identify and classify different plant (i.e. micro- and macroalgae) and animal (i.e. gastropods and other sessile fauna) species. Unlike these methods, the imagery provided by our technique is able to capture fine-scale details in the structure, texture and pattern in the imagery (see for example Figure 11), owing to the high-spatial resolution, which could be used instead to automatically classify objects in the intertidal zone through the use of methods in semi-supervised machine learning and object-based image analysis [53]. For example, the colour and spectra information of cunjevoi or algae such as *Homosira* produce a uniquely identifiable texture and pattern in high-resolution imagery, owing to their physical structures, that could be used as classification features that would not be detected via traditional, lower-resolution remote sensing methods.

### Potential Uses in Monitoring and Environmental Impact Assessment

To reliably detect human and environmental impacts on plant and animal populations it is necessary to sample populations at several times and at appropriate spatial resolutions before and after an impact (see [10]). Often, it is not possible to do this either for logistical or cost constraints, thus reducing the reliability of the detection of change. Our method provides the potential for distinguishing natural changes such as seasonality from human impacts such as construction, pollution or climate change in intertidal environments in a statistically robust way; the low-cost and logistical simplicity of our data collection procedure lends itself to frequent data collection, and by collecting data at fine-scales over broad areas and with full coverage of the landscape, potentially allows for precise spatial registration of data collected over multiple surveys.

### Application to Studying Intertidal Mudflats

The method presented here is not limited to rocky intertidal substrata but could also be used for various applications on intertidal mud flats. Conditions on intertidal mud flats can change over an interval of a few minutes - large changes in sediment properties can occur including dewatering of the sediment and the migration of microphytobenthos to the surface [54]. These changes can have significant impacts upon sediment stability over the course of a single tidal cycle. Due the dynamic nature of intertidal mudflats combined with the relatively slow pace of conventional field sampling make it impossible to make measurements of sediment properties across space that are truly independent of changes in time. Our method, by enabling large amounts of data to be acquired in a snapshot of time, at specific times in the tidal cycle, enables independent measurements to be made. Applications include the determination of impacts of structures on sediment properties or the effects of spillage of contaminants e.g. algicides, pesticides or fertiliser [55] on phytobenthos.

## Conclusions and Future Work

In this paper, we have described a photogrammetric/mapping procedure that was developed for constructing high-resolution, 3D, photo-textured terrain models of intertidal areas using multiple low-altitude images collected from a consumer-grade digital camera suspended by a kite platform. Dynamic intertidal ecosystems by their nature can change rapidly at the scale of minutes to years making it almost impossible to acquire data that describe changes that occur spatially independently of temporal changes using field-based sampling. The methods presented acquire colour and topographic information across a hierarchy of spatial scales in a very small time interval, enabling changes in spatial distributions of assemblages to be determined independently of temporal changes, and at resolutions not achievable by traditional remote sensing platforms (such as satellites and manned-aircraft).

Ongoing and future work is focusing on three main areas. The first area is to develop methods for registering map data collected across multiple surveys. In some cases, the visual features (i.e. SIFT) used to register images within a survey will be suitable for this process, however the stability of these features is known to diminish when the time between images increases owing to small-scale changes in the imaged surface. Instead, current work is focussing on using robust means for multi-survey registration commonly used in remote sensing and medical imaging, such as mutual information [56], [57], to align multi-temporal datasets. The second area of future work involves the investigation of object based image analysis and machine learning algorithms for semi-supervised classification of dominant coverage types, such as different macroalgae, in the intertidal zone. This type of automated analysis could complement the mapping and reconstruction algorithms presented in this paper, providing useful information for ecologists and marine park managers without the need for laborious manual interpretation of the large quantity of image data. The third area of future work is focussing on using tethered kites and balloons as a potential platform for carrying low-cost, hyperspectral imagers or spectroradiometers to provide higher spectral resolution information than provided by the consumer-grade cameras presented in this study. Accurate spectroradiometers are becoming more accessible as technology improves, and the combination of high resolution photography and single-point spectroradiometer measurements, via cross-sensor calibration and the use of the photogrammetry techniques discussed in this paper, could provide a means to producing spatially-registered, airborne hyperspectral maps, at a lower-cost than via manned aircraft imaging systems.
